# Purple bamboo salt has anticancer activity in TCA8113 cells *in vitro* and preventive effects on buccal mucosa cancer in mice *in vivo*

**DOI:** 10.3892/etm.2012.848

**Published:** 2012-12-04

**Authors:** XIN ZHAO, XIAOXIAO DENG, KUN-YOUNG PARK, LIHUA QIU, LIANG PANG

**Affiliations:** 1 Department of Biological and Chemical Engineering, Chongqing University of Education, Chongqing 400067, P.R. China;; 2 Department of Food Science and Nutrition, Pusan National University, Busan 609-735, Republic of Korea;; 3 Department of General Knowledge, Chongqing University of Education, Chongqing 400067;; 4 Department of Oral and Maxillofacial Surgery, The Affiliated Hospital of Stomatology,Chongqing Medical University, Chongqing 401147, P.R. China

**Keywords:** purple bamboo salt, buccal mucosa cancer, Institute of Cancer Research mice, U14 squamous cell carcinoma cells, metastasis

## Abstract

Bamboo salt is a traditional healthy salt known in Korea. The *in vitro* anticancer effects of the salt were evaluated using a 3-(4,5-dimethyl-2-thiazolyl)-2,5-diphenyltetrazolium bromide (MTT) assay in TCA8113 human tongue carcinoma cells. At 1% concentration, the growth inhibitory rate of purple bamboo salt was 61% higher than that of sea salt (27%). Apoptosis analysis of the cancer cells was carried out using 4,6-diamidino-2-phenylindole (DAPI) staining to investigate the mechanism of the anticancer effects in tongue carcinoma cells. Purple bamboo salt induced a stronger apoptotic effect than sea salt. An Institute of Cancer Research (ICR) mouse buccal mucosa cancer model was established by injecting mice with U14 squamous cell carcinoma cells. Following injection, the wound at the injection site was smeared with salt samples. It was observed that the tumor volumes for the group treated with purple bamboo salt were smaller than those from the sea salt treatment and control groups. The sections of buccal mucosa cancer tissue showed that canceration in the purple bamboo salt group was weaker compared with that in the sea salt group. Similar results were observed in the lesion section of the cervical lymph. Using reverse transcription-polymerase chain reaction (RT-PCR) and western blotting, the purple bamboo salt group demonstrated an increase in Bcl-2-associated X protein (Bax) and a decrease in B cell lymphoma-2 (Bcl-2), inducible nitric oxide synthase (iNOS) and cyclooxygenase-2 (COX-2) expression, compared with the sea salt and control groups. The results demonstrated that purple bamboo salt had improved *in vivo* buccal mucosa cancer preventive activity compared with sea salt in mice.

## Introduction

Bamboo salt, as a functional food, originated in Korea. Approximately 1,000 years ago, Korean doctors and monks began creating medical salt. The bamboo salt was prepared by putting sea salt into a case made from young bamboo, which had grown for just 3 years. The two ends were sealed using natural red clay and the bamboo case was baked at 1,000–1,500°C (1,2), using pine as the fuel. In ancient times, the bamboo salt was baked only 2-3 times. The salt was then used as a special medical treatment. Eventually, it was identified that bamboo salt acted at its highest medical efficacy if it was baked at least 9 times. The 9-times-baked bamboo salt was named purple bamboo salt. Additionally, it was observed that if bamboo salt is completely melted, the toxic characteristic of the salt disappeared.

Currently, bamboo salt is one of the most well-known traditional medical treatments, not only in Korea but also in a number of other Asian countries (3,4). Bamboo salt contains >70 essential minerals and micronutrients. Pharmaceutical scientists are researching the special therapeutic qualities of bamboo salt, including its anticancer and antiviral effects (5). The researchers noted that bamboo salt demonstrates anti-inflammatory and antioxidant effects and that it may also be used as bamboo salt toothpaste for dental disease and oral hygiene (6,7).

Buccal mucosa cancer is the most common cancer of the oral cavity (8). In the present study, the cancer preventive effect of purple bamboo salt was evaluated using a mouse model of buccal mucosa cancer. The bamboo salt was shown to enhance anti-cancer activities and the anti-metastatic effect in mice. As a functional food, purple bamboo salt demonstrated oral health benefits in mice.

## Materials and methods

### Preparations of bamboo salt

The purple bamboo salt (9-times-baked bamboo salt) and sea salt were provided by Taesung Food Company (Gochang, Jeonbuk, Korea). The salts were dissolved in distilled water.

### Cell preparation

TCA8113 human tongue carcinoma cells obtained from Shanghai Institute of Biochemistry and Cell Biology (Shanghai, China) and U14 squamous cell carcinoma cells obtained from Chinese Academy of Medical Sciences (Beijing, China) were used in this study. The cancer cells were cultured in RPMI-1640 medium (Welgene Inc., Daegu, Korea) supplemented with 10% fetal bovine serum (FBS) and 1% penicillin-streptomycin (Gibco-BRL, Grand Island, NY, USA) at 37°C in a humidified atmosphere with 5% CO2 (incubator model 311 S/N29035; Forma, Waltham, MA, USA). The medium was changed 2 or 3 times a week (5).

*In vitro* cultured U14 cells (5×10^6^/mouse) were injected into the abdominal cavity of 7-week-old female Institute of Cancer Research (ICR) mice. After 1 week, the carcinoma ascites were collected and diluted in sterile saline to a concentration of 1×10^7^/ml.

### 3-(4,5-Dimethyl-2-thiazolyl)-2,5-diphenyltetrazolium bromide (MTT) assay

The anticancer effects of purple bamboo salt were assessed by an MTT assay. The TCA8113 human tongue carcinoma cells (180 *μ*l) were seeded onto a 96-well plate (2×10^4^ cells/ml/well). The specimen (20 *μ*l) was added to the vessel to be cultured at 37°C, 5% CO_2_ for 48 h. MTT solution (200 *μ*l, 5 mg/ml) was added and the cells were cultured for a further 4 h under the same conditions. After removing the supernatant, 150 *μ*l dimethylsulfoxide (DMSO) was added to each well and mixed for 30 min. Finally, the absorbance of each well was measured using an enzyme-linked immunosorbent assay (ELISA) reader (model 680; Bio-Rad, Hercules, CA, USA) at 540 nm (9).

### Nuclear staining with 4,6-diamidino-2-phenylindole (DAPI)

Untreated control and cells treated with purple bamboo salt were harvested, washed with phosphate-buffered saline (PBS) and fixed with 3.7% paraformaldehyde (Sigma, St. Louis, MO, USA) in PBS for 10 min at room temperature. The fixed cells were washed with PBS and stained with DAPI (1 mg/ml; Sigma) solution for 10 min at room temperature (10). The cells were washed 2 more times with PBS and examined under a fluorescence microscope (BX50; Olympus, Tokyo, Japan).

### Induction of buccal mucosa cancer

Female ICR mice (n=40, 6 weeks old) were purchased from the Experimental Animal Center of Chongqing Medical University (Chongqing, China). They were maintained in a temperature-controlled (temperature, 25±2°C; relative humidity, 50±5%) facility with a 12 h light/dark cycle and had unlimited access to a standard mouse chow diet and water.

To investigate the preventive effects of the two salts against buccal mucosa cancer induced by injecting U14 cells into the mice, the animals were divided into 4 groups with 10 mice in each. The experimental design was as follows: the mice in the two salt sample groups were smeared with the purple bamboo salt and sea salt solutions (20%) on the buccal mucosa every 12 h for 14 days. The control and salt sample groups were then inoculated with 0.05 ml cancer cell suspension (1×10^7^/ml) on the buccal mucosa. The salt samples continued to be smeared on the buccal mucosa of the mice every 12 h. The mice were sacrificed 14 days later and their tumor volumes and lymph node metastasis rates were determined (11).

### Histological grading of buccal mucosa cancer

Buccal mucosa and lymph node tissues were removed and embedded into paraffin for histological analysis with hematoxylin and eosin (H&E) staining. Buccal mucosa cancer was graded as follows: i) well-differentiated carcinoma: cells resemble the adjacent benign squamous epithelium; ii) moderately differentiated carcinoma: cells form large anastomosing areas in which keratin pearls are formed, they are not numerous and the main component consists of cells with pronounced cytonuclear atypia and iii) poorly differentiated carcinoma: cells have lost the majority of their squamous epithelial characteristics and architecture (12).

### Reverse transcription-polymerase chain reaction (RT-PCR) analysis of Bcl-2-associated X protein (Bax), B cell lymphoma-2 (Bcl-2), inducible nitric oxide synthase (iNOS) and cyclooxygenase-2 (COX-2) mRNA expression

Total RNA was isolated using TRIzol reagent (Invitrogen, Carlsbad, CA, USA) according to the manufacturer's instructions. RNA was digested with RNase-free DNase (Roche, Basel, Switzerland) for 15 min at 37°C and purified using an RNeasy kit (Qiagen, Hilden, Germany) according to the manufacturer's instructions. cDNA was synthesized from 2 *μ*g total RNA by incubation at 37°C for l h with avian myeloblastosis virus (AMV) reverse transcriptase (GE Healthcare, Uppsala, Sweden) with random hexanucleotide, according to the manufacturer's instructions. Primers used to specifically amplify the genes were as follows: forward, 5′-AAG CTG AGC GAG TGT CTC CGG CG-3′ and reverse, 5′-CAG ATG CCG GTT CAG GTA CTC AGT C-3′ for Bax; forward, 5′-CTC GTC GCT ACC GTC GTG ACT TGG-3′ and reverse, 5′-CAG ATG CCG GTT CAG GTA CTC AGT C-3′ for Bcl-2; forward, 5′-AGA GAG ATC GGG TTC ACA-3′ and reverse, 5′-CAC AGA ACT GAG GGT ACA-3′ for iNOS; forward, 5′-TTA AAA TGA GAT TGT CCG AA-3′ and reverse, 5′-AGA TCA CCT CTG CCT GAG TA-3′ for COX-2. The internal control gene glyceraldehyde 3-phosphate dehydrogenase (GAPDH) was amplified with the following primers: forward, 5′-CGG AGT CAA CGG ATT TGG TC-3′ and reverse, 5′-AGC CTT CTC CAT GGT CGT GA-3′. Amplification was performed in a thermal cycler (Eppendorf, Hamburg, Germany) with 29 Bax cycles, 34 Bcl-2 cycles, 25 iNOS, COX-2 and GAPDH cycles of denaturation. The amplified PCR products were run in 1.0% agarose gels and visualized by ethidium bromide (EtBr) staining (13).

### Western blot analysis

Total protein was obtained with RIPA buffer as described by Kim *et al*(14). Protein concentrations were determined with a Bio-Rad protein assay kit. For western blot analysis, aliquots of the lysate containing 30–50 *μ*g protein were separated by sodium dodecyl sulphate-polyacrylamide gel electrophoresis (SDS-PAGE) and then electrotransferred onto a nitrocellulose membrane (Schleicher and Schuell, Keene, NH, USA). The membranes were subjected to immunoblot analysis and proteins were visualized by an enhanced chemiluminescence (ECL) method (GE Healthcare). The cell lysates were separated by 12% SDS-PAGE, transferred onto a polyvinylidene fluoride membrane (GE Healthcare), blocked with 5% skimmed milk and hybridized with primary antibodies (diluted 1:1,000). Antibodies against Bax, Bcl-2, iNOS and COX-2 were obtained from Santa Cruz Biotechnology, Inc. (Santa Cruz, CA, USA), then incubated with the horse-radish peroxidase-conjugated secondary antibody (Santa Cruz Biotechnology, Inc.) for 1 h at room temperature. Blots were washed 3 times with PBS/Tween-20 (PBS-T) and then developed by enhanced chemiluminescence (Amersham Life Science, Arlington Heights, IL, USA).

### Statistical analysis

Data are presented as mean ± standard deviation. Differences between the mean values for the individual groups were assessed by a one-way analysis of variance (ANOVA) with Duncan's multiple range tests. P<0.05 was considered to indicate a statistically significant difference. The SAS v9.1 statistical software package (SAS Institute Inc., Cary, NC, USA) was used for the analysis.

## Results

### Growth inhibitory effects of salt samples on TCA8113 cells

The anticancer effects of purple bamboo salt and sea salt on TCA8113 human tongue carcinoma cells were investigated. The growth inhibitory rates of TCA8113 cells treated with 0.5% salt samples were 12 and 35% for sea salt and purple bamboo salt, respectively. At 1.0%, the growth inhibitory rates of cells treated with these reagents were 27 and 61%, respectively (P<0.05). It was clear that the anticancer effect of purple bamboo salt on the TCA8113 cells was stronger than that of sea salt (Table I).

### Induction of apoptosis by salt samples on TCA8113 cells

**Figure 1.**
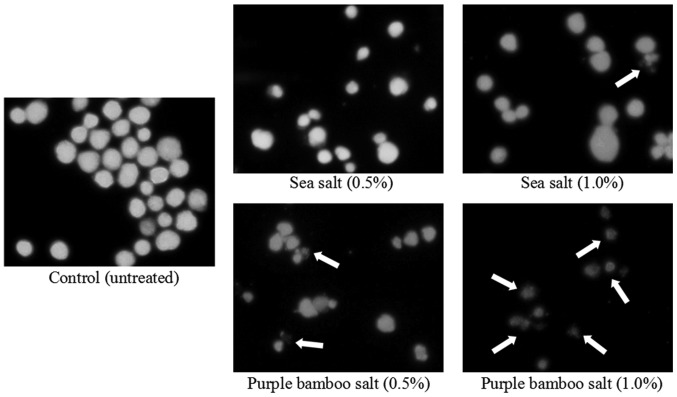
Induction of apoptosis in TCA8113 human tongue carcinoma cells by treatment with salt samples. Cells were incubated with samples of salt for 48 h and then stained with DAPI. After 10 min incubation at room temperature, the cells were washed with PBS and photographed with a fluorescence microscope using a blue filter. Magnification, x400. DAPI, 4,6-diamidino-2-phenylindole; PBS, phosphate-buffered saline.

To further determine whether the growth inhibitory activity of purple bamboo salt in the TCA8113 cells was related to the induction of apoptosis, chromatin condensation was analyzed by fluorescent microscopy using the DNA binding fluorescent dye DAPI (Fig. 1). In TCA8113 cells, which normally contain nuclei with homogeneous chromatin distribution, treatment with salt samples (1.0%) induced chromatin condensation and nuclear fragmentation, suggesting the presence of apoptotic cells. Chromatin condensation and formation of apoptotic bodies, which are characteristic of apoptosis, were observed in the cells cultured with purple bamboo salt; however, these were not identified in the cells treated with sea salt. These results suggest that purple bamboo salt is more effective for inducing the condensation and formation of apoptotic bodies than sea salt.

### Tumor volumes and lymph node metastasis rates

Buccal mucosa cancer was induced by injecting U14 cells into mice. After 14 days, the mice in all groups presented carcinogenesis. The tumor volumes of buccal mucosa tissues were measured. The tumor volumes for the control, sea salt and purple bamboo salt groups were 12.4, 12.0 and 7.2 mm^3^, respectively (Table II). There were 5 mice demonstrating lymph node metastasis in the control group, 4 in the sea salt group and 2 in the purple bamboo salt group. Consequently, the lymph node metastasis rate was 50, 40 and 20%, respectively. These results demonstrate that purple bamboo salt is more effective than sea salt in impeding carcinogenesis, proliferation and metastasis.

### Histopathology of buccal mucosa tissues

**Figure 2.**
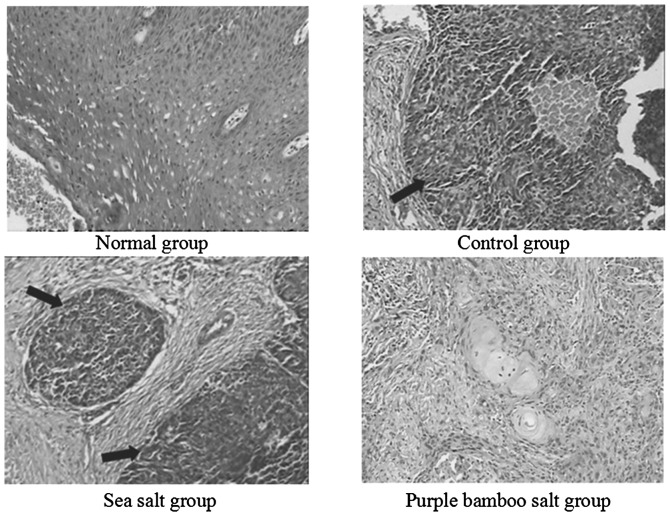
Histology of buccal mucosa tissues induced by injecting U14 squamous cell carcinoma cells into mice (hematoxylin and eosin staining; magnification, x100).

Histological changes in the buccal mucosa of mice injected with U14 cells were examined by H&E staining. The histologic tissue sections of mice in the normal group demonstrated normal histological morphology of squamous epithelium tissue. Histopathological evaluation revealed indications of buccal mucosa cancer in both groups receiving U14 cells (Fig. 2). The sections from mice in the control and sea salt groups showed that the tissue lost its squamous epithelial characteristics and architecture; however, the tissues from the sea salt group had some intracellular bridging between the normal squamous cells. The histopathology sections indicated that the mice in the control and sea salt groups developed poorly differentiated carcinoma (grade III) and the control group experienced more serious carcinogenesis. The tissue sections of purple bamboo salt group looked less like typical squamous epithelium. The tumor cells remained in nests and there were numerous larger, eosinophilic, polygonal cells that were trying to layer themselves in a squamous-like fashion. However, for the purple bamboo salt group, the overall resemblance to typical squamous epithelium was less striking (grade II). From these sections, purple bamboo salt demonstrated a preventive effect against buccal mucosa cancer.

**Figure 3.**
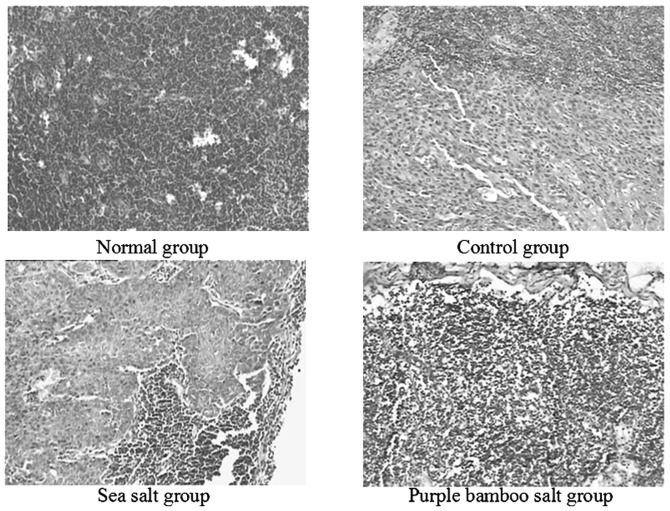
Histology of buccal mucosa cancer cervical lymph node metastasis (hematoxylin and eosin staining; magnification, x100).

The lymph node sections from the control group exhibited a large area with liquefaction necrosis of the lymph node (Fig. 3). Marked liquefaction necrosis was also identified in the sea salt group, whereas only a small amount of liquefaction necrosis was observed in the purple bamboo salt group. These results demonstrate that purple bamboo salt is more effective than sea salt in preventing lymph node metastasis.

### Gene expressions of Bax, Bcl-2, iNOS and COX-2 in buccal mucosa tissues

**Figure 4.**
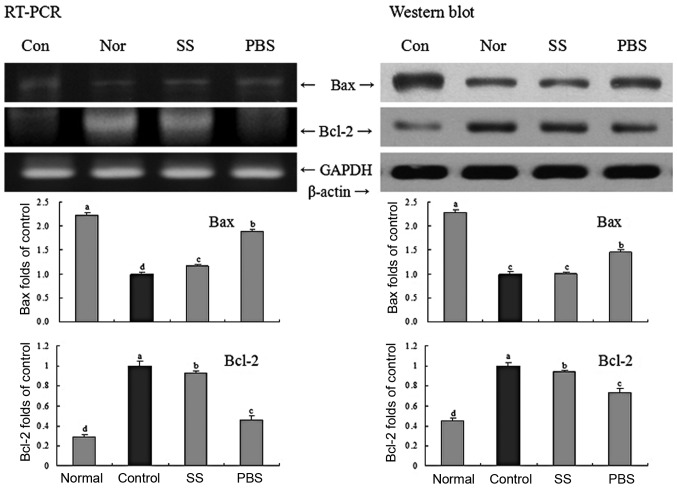
Effects of salt samples on mRNA and protein expressions of Bax and Bcl-2 in buccal tissues. Con, control group; Nor, normal group; SS, sea salt group and PBS, purple bamboo salt group. The intensity of each band was measured with a densitometer and expressed as fold rate of control. Fold rate: target expression/GAPDH (β-actin) x control numerical value (control fold ratio: 1). ^a–d^Means with the different letters are significantly different (P<0.05) by Duncan's multiple range test. RT-PCR, reverse transcription-polymerase chain reaction; Bax, Bcl-2-associated X protein; Bcl-2, B cell lymphoma 2; GAPDH, glyceraldehyde 3-phosphate dehydrogenase.

**Figure 5.**
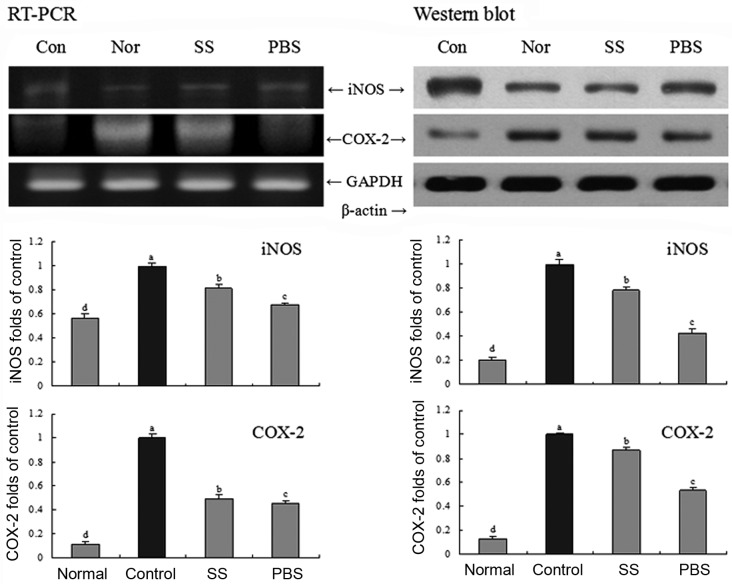
Effects of salt samples on the mRNA and protein expression of iNOS and COX-2 in buccal tissues. Con, control group; Nor, normal group; SS, sea salt group and PBS, purple bamboo salt group. The intensity of each band was measured with a densitometer and expressed as fold rate of control. Fold rate: gene expression/GAPDH (β-actin) x control numerical value (control fold ratio: 1). ^a–d^Means with different letters are significantly different (P<0.05) by Duncan's multiple range test. RT-PCR, reverse transcription-polymerase chain reaction; iNOS, inducible nitric oxide synthase; COX-2 cyclooxygenase-2; GAPDH, glyceraldehyde 3-phosphate dehydrogenase.

To determine the protective mechanisms against buccal mucosa cancer, the expression levels of Bax and Bcl-2 in buccal mucosa tissues were determined by RT-PCR and western blotting. As shown in Fig. 4, in the group treated with purple bamboo salt, the pro-apoptotic Bax and the anti-apoptotic Bcl-2 showed significant changes. Accordingly, the results suggested that the purple bamboo salt induced apoptosis in buccal mucosa tissues via a Bax- and Bcl-2-dependent pathway. Thus, apoptosis induction in the purple bamboo salt group was related to an increase in Bax and a decrease in Bcl-2 in terms of mRNA and protein expression compared with the sea salt and control groups. RT-PCR and western blot analysis were also conducted to investigate whether the inhibitory effect of salts on inflammation were due to gene regulation of inflammatory mediators, including iNOS and COX-2. COX-2 and iNOS are important enzymes that mediate inflammatory processes. As shown in Fig. 5, these inflammatory mediators were barely detectable in the normal group. However, the control group demonstrated a sharp increase in mRNA and protein expression levels. In the purple bamboo salt group, the results showed a significant decrease in the mRNA and protein expression levels of COX-2 and iNOS. It was observed that higher decreases in mRNA and protein expression were related to improved anti-inflammatory effects.

## Discussion

Bamboo salt has been used as a folk medicine; however, scientific data on the effects of this salt are lacking. Nine-times-baked bamboo salt, also called purple bamboo salt since the color changes to purple following the baking process, has high concentrations of iron, silicon and potassium compared with those in crude salt (15). This salt has been previously reported to have various therapeutic effects on numerous pathological conditions, including inflammation, viral diseases, diabetes and cancer (16). The bamboo salt is also known to have *in vitro* anticancer effects on HT-29 colon cancer cells (5). We also investigated the *in vitro* anticancer effect of purple bamboo salt in the current study.

Programmed cell death (apoptosis) is a conserved, natural mechanism for the removal of redundant and unwanted cells during normal development. Whether apoptosis is the cause or the consequence of drug-induced cell death remains to be established (17). The importance of apoptosis is widely recognized in numerous fields of medicine and is currently one of the most popular subjects in biomedical research. Apoptosis is a fundamental cell event and the understanding of its mechanisms will shed light on its future use in tumor diagnosis and therapy.

Metastasis is defined as the spread of cancer cells from one organ or area to another adjacent organ or location (18,19). It is thought that malignant tumor cells have the capacity to metastasize. Cancer occurs after cells in a tissue are genetically damaged in a progressive manner, resulting in cancer stem cells possessing a malignant phenotype. After the tumor cells come to rest in another site, they penetrate the vessel walls, continue to multiply and eventually form another tumor.

Histopathology is an important tool in anatomical pathology, since accurate diagnosis of cancer usually requires histopathological examination of samples. Histopathology is an important clinical standard for the diagnosis of oral cancer (20).

In a healthy cell, the outer mitochondrial membrane expresses the anti-apoptotic protein Bcl-2 on its surface (21). Bcl-2 is bound to a protein named Apaf-1. Cell internal damage causes Bcl-2 to release Apaf-1 (22) and a Bcl-2-related protein (Bax) penetrates the mitochondrial membrane, causing cyto-chrome *c* to leak out into the cytosol (23). In the current study, purple bamboo salt demonstrated greater *in vivo* anticancer activity than sea salt, as was observed using RT-PCR and western blotting of buccal mucosa cancer tissues. The anti-cancer mechanisms of purple bamboo salt include the induction of apoptosis by increasing the number of apoptotic bodies and by regulating the expression levels of the apoptosis-related Bax and Bcl-2 mRNA and proteins.

Improper upregulation of COX-2 and/or iNOS has been associated with the pathophysiology of certain types of human cancers, as well as inflammatory disorders. Since inflammation is closely linked to tumor promotion, substances with potent anti-inflammatory activities are anticipated to exert chemopreventive effects on carcinogenesis, particularly in the promotion stage (24).

Accordingly, purple bamboo salt is expected to contribute to the prevention of buccal mucosa cancer. The potassium, calcium, magnesium and iron contents in bamboo salts are higher than those in purified and solar salts. Additionally, bamboo salt baked for longer periods of time contains more minerals (25). Increased levels of these minerals in the salt are important for enhancing the anticancer effects (26). Bamboo salt also exhibits a higher reduction potential; this may be due to the fact that this type of salt contains more hydroxyl (OH) ions than purified or solar salts (27). Overall, bamboo salt seems to have more potent anticancer and anti-inflammatory effects since it contains higher levels of minerals and more OH ions.

In the current study, we employed *in vitro* and *in vivo* anticancer experimental methods, including MTT assay, histo-pathology assay and RT-PCR mRNA and western blotting protein expression assays to determine the anticancer and anti-inflammatory effects of purple bamboo salt. In addition, we obtained results demonstrating that the anti-metastatic effect of purple bamboo salt is greater than that of sea salt. In conclusion, the increased mineral contents, and other phytochemicals are important functional compounds that may increase the buccal mucosa cancer preventive effect of purple bamboo salt.

## Figures and Tables

**Table I.** Inhibition of the growth of TCA8113 human tongue carcinoma cells by salt samples as evaluated by a 3-(4,5-dimethyl-2-thiazolyl)-2,5-diphenyltetrazolium (MTT) assay.

Treatment	OD_540_ (inhibition rate)
Control (untreated)	0.815±0.006^a^
Sea salt	
0.5%	0.717±0.011 (12)^b^
1.0%	0.595±0.007 (27)^c^
Purple bamboo salt	
0.5%	0.530±0.009 (35)^d^
1.0%	0.318±0.009 (61)^e^

a P<0.05 vs. the control group;

b P<0.05 vs. the sea salt (0.5%) group;

c P<0.05 vs. the sea salt (1.0%) group;

d P<0.05 vs. the purple bamboo salt (0.5%) group;

e P<0.05 vs. the purple bamboo salt (1.0%) group. OD, optical density.

**Table II.** Tumor volumes and lymph node metastasis rates of mice smeared with salt samples.

Group	Normal	Control	Sea salt	Purple bamboo salt
Tumor volume (mm^3^)	0	12.4±0.6	12.0±0.5	7.2±0.2
Lymph node metastasis rate^a^	0	5/10 (50%)	4/10 (40%)	2/10 (20%)

a Number of lymph node metastases/total number.
